# Cavity Linings

**Published:** 1903-12-15

**Authors:** B. L. Thorpe


### CAVITY LININGS.

BY B. L. THORPE, D.D.S.

Read before the Missouri Dental Society.

Before attempting cavity lining, the rubber dam should be applied; the operator, being thoroughly familiar with several of the many materials, will choose the one indicated for the case in hand. The requisites for a cavity lining are, viz: *hardness, translucency, non-conductivity and slightly antiseptic properties.*

The admissible insulating cavity lining materials are:

Cements: Oxychloride of zinc, oxyphosphate of zinc, oxysulphate of zinc.

Gutta-percha: Chlora-percha or similar solutions.

Gold: Gold and platinum mats, No. 2, non-cohesive foil, gold and tin foil.

Proprietary remedies: Cavitine, Fletcher's carbolized resin, Weston's non-irritant cement, Wessel's non-conducting cavity lining, balsamo del deserto, auro-percha, Caulk's and Gilbert's lining, Dr. L. A. Young's oleo-percha.

Miscellaneous material: Quill, waxed paper, asbestos felt foil.

Solution of the resinous gums, dissolved in ether, chloroform or alcohol: Resin of pinus silvestris, copal, Canada balsam, sandarach, damar, mastic, amber, etc.

*Cement.*—The zinc plastics preserve by their stimulating qualities, which prevent recurrence of decay, and promote deposit of secondary dentine in the pulp-chamber; especially is this true of zinc chloride, which, however, if applied directly to the dentine in close proximity to the pulp, will cause its destruction. This is the general and foremost objection to this valuable material, which seems little appreciated by the younger dentists of to-day, but it is, without doubt, the best barrier to bacteria of all the materials at our command. In lining cavities, it should be preceded—in fact, all cavity linings should be preceded with a thin coat of some of the resinous varnishes, which occupy an infinitesimally small space and which prevent the escharotic action of the cement fluid on the pulp.

As stated before, the application of chloride of zinc in deep-seated cavities is dangerous. Its affinity for water induces irritation, which is followed by devitalization of the pulp, leaving that organ in a mummified condition. The cauterant properties of the liquid may be greatly modified by diluting the solution with an equal amount of water, and mixing the powder into a stiff paste.

After excavation and sterilization of the cavity, the oxychloride is forced against the cavity walls with a pledget of punk or bibulous paper, which absorbs the surplus moisture and adapts it to the cavity walls. This preparation sets slowly, but can be hastened by the application of a hot-air blast from the chip-blower. After fifteen minutes the margins should be cleaned of the oxychloride, and the patient dismissed until the next appointment, when the metal filling may be completed. While "extension for prevention" is the fad of to-day, many teeth of pure structure, with weak walls, may be lined and saved for years with an oxychloride lining.

*Zinc Phosphate.*—Before the application of zinc phosphate, to safely protect the dentinal walls from contact with the acid phosphate of zinc, the cavity must be protected with a coat of some of the resinous varnishes, or a coating of gutta or chlora-percha. The *modus operandi* of lining the cavity with this cement is similar to the one outlined for zinc chloride.

Zinc phosphate is not a good cavity lining; it does not possess the antiseptic or stimulating properties of zinc chloride. Phosphate of zinc linings, in too close proximity to the pulp, invariably result in its death from the corroding action of the acid, *and not*, as it has wrongfully been attributed, *to the presence of arsenical compounds in the cement powder.*

*Oxysulphate of Zinc.*—Zinc sulphate cement, better known as Fletcher's artificial dentine, has been advocated for the above purposes. It is non-irritating, as the liquid of this cement comprises a simple solution of gum arabic in water. It possesses no antiseptic properties.

*Gutta-percha.*—Gutta-percha is not an ideal cavity lining, however, it can be applied successfully by first using the varnish and then adapting the warmed material to the walls while the varnish is in a sticky state. The gutta-percha should be covered with either of the cements. This prevents the dragging of the materials from the cavity walls. *Metal fillings should never be placed over gutta-percha; a layer of cement should always intervene between them* to prevent expansion which is followed by the displacement of the fillings.

*Chlora-percha.*—Chlora-percha is hardly ever indicated, on account of the shrinkage and shriveling of the material on the evaporation of the chloroform.

*Gold and Platinum Mats, No. 2.*—A lining as applied by late the Dr. William N. Morrison, of St. Louis, make an excellent lining for cavities to be filled with alloy, where the material is likely to discolor the tooth. With cavity prepared, the side of the mat that comes in contact with the tooth-wall varnished, with bibulous paper and ball burnisher press the mat to place; when varnish is dry, varnish entire cavity and mat, and pack alloy. Non-cohesive foil and gold and tin foil, quill, waxed paper and asbestos felt foil should be applied in the same manner.

*Resinous Varnishes.*—In sensitive cavities, no material so nearly fills the requirements of an ideal lining as some of the various varnishes made from some of the resins. They furnish a non-conducting and impermeable film covering the dentinal walls, occupying small space, sealing the tubuli and combining mechanically with the tooth structure, thus forming close adhesion between the plastic and cavity walls. The best varnish for such a purpose is a solution of the resin of pinus silvestris, 2 drams dissolved in one dram absolute alcohol, as recommended by Dr. A. C. Hewett, of Chicago. This resin is a product of the Scotch fir-tree, and is sold in music stores, being commonly known as "fiddle-bow rosin." This, like all gums of its kind, possesses antiseptic properties from the essential oils contained therein. This make a hard, glossy coating; when applied to a dry, sensitive cavity and dried, acts as an instant insulator of thermal shock.

*Copal.*—Another adhesive and antiseptic cavity varnish is that of gum copal dissolved in ether, alcohol or chloroform, to which may be added about 10 percent hydronapthol. Other varnishes of merit, are sandarach, Canada balsam, mastic, damar, etc., but the pinus silvestris solution is by far superior to all other resins. In cases where a filling of gold and alloy approximate in adjoining cavities, viz: in vital bicuspids or molar teeth, a galvanic current is often produced; the electrolytic action may be greatly modified by varnishing the cavity before the fillings are inserted.

*Summary of Advantages.*—The use of a suitable insulating material will prove the advantages of cavity linings, to seal the tubuli and cover the dentine with a non-irritating and non-conducting material that cuts off electrical action between fillings and dentine, and prevents escharotic action of the zinc cements upon the pulp; prevents recurrent decay and penetration of moisture, preserving the tooth's translucency, when it is necessary to insert a material that colors, giving longer service of filling and comfort from thermal shock, and preventing the need of cutting away the tooth structure for extension.—*Western Journal.*

